# The Correlation between Organizational (School) Climate and Teacher Job Satisfaction—The Type of Educational Institution Moderating Role

**DOI:** 10.3390/ijerph19116520

**Published:** 2022-05-27

**Authors:** Wojciech Otrębski

**Affiliations:** Institute of Psychology, The John Paul II Catholic University of Lublin, Al. Racławickie 14, 20-950 Lublin, Poland; otrebski@kul.pl; Tel.: +48-516-120-094

**Keywords:** inclusive education, school climate, job satisfaction, work affect, mainstream or special schools

## Abstract

The quality of work and task implementation in educational institutions to a large extent depends on the personnel of those institutions. This is particularly true in the case of teaching pupils with special educational needs. The climate of education and learning and job satisfaction depend on teachers themselves. The purpose of this study was to verify the extent to which the type of educational institution (a mainstream or special school) moderates the correlation between teachers’ subjective evaluation of the organizational climate, their job satisfaction, and work-related emotions. The study involved 214 teachers representing all levels of school education in Poland, i.e., primary, middle, and high schools attended by pupils with special educational needs. Half of the teachers worked in mainstream schools and the other half—in special schools. The study used: The Organizational Climate Questionnaire by Litwin and Stringer; The Satisfaction with Job Scale; and The Work Affect Scale. The results clearly suggest that the teachers’ perception of the organizational climate is a strong predictor of their job satisfaction and work-related emotions. A positive climate is associated with high satisfaction and good emotions (enthusiasm and comfort) while a negative climate co-occurs with low satisfaction and bad emotions (anxiety and depression). The organizational type of institution (mainstream or special school) does not significantly affect the above predictive attribute of the organizational climate.

## 1. Introduction

Disability and the resulting special needs of an individual are the focus of interest of numerous social, medical, and educational services. The service that tries to integrate all the support provided to persons with disabilities is complex rehabilitation. It is understood as a set of activities undertaken in order to secure all the current needs of a person with disabilities (medical, educational, professional, and social aspects). The main goal of complex rehabilitation is to improve the quality of functioning, activity, and participation of every person with disabilities and of their environment [1].

Considering only the educational aspect of complex rehabilitation, it is noted that in the last decade, a major challenge for many educational systems in Europe has been to ensure high quality education in mainstream schools, where abled and disabled pupils pursue their educational needs together [2,3]. Countries making efforts to strive to maximize the percentage of pupils with disabilities in mainstream schools receive significant support from the European Agency for Special Needs and Inclusive Education [4]. According to the Polish Ministry of Education and Sciences, in 2022, more than 70% of pupils with special educational needs in primary and secondary education attended inclusive schools and the other 30% were enrolled in special schools [5].

This trend in the European system of education indicates the priority directions for scientific research and analyses needed to support countries in the implementation of measures within the model of high-quality education for all learners, including typical pupils and those with disabilities. In response to this need, a number of studies have been undertaken to identify various (institutional and personal) indicators of the readiness of the mainstream educational institutions to provide high quality education for all learners. This report presents the results of some of those studies that explored the correlation between the climate in an organization (school) and job satisfaction and the emotions experienced by teachers who work in that organization. It also analyses how the type of an educational institution (a mainstream or special school) moderates that correlation.

### Theoretical Background

For many years, the results of studies conducted by psychologists among teachers/educators have highlighted the stress generated by this profession, the same as other professions that involve direct contacts with the client. The worst consequence of prolonged stress is occupational burnout [6]. Aware of this regularity, researchers started to seek the determinants of the burnout syndrome. Most of them point to two groups of factors that may contribute to occupational burnout: personal variables (socio-demographic and personality characteristics) ([7], p. 87) and institutional variables (related to work and the organization) ([8], p. 230).

Undoubtedly, a teacher who is qualified mainly to work with a typical pupil in a mainstream school may experience a high level of stress when having to dramatically adjust their teaching and work methods to the special educational needs of a pupil [9,10]. The way a teacher copes with such situations largely depends on their individual resources and the resources available within the organization. In order for an educational institution to be inclusive, i.e., to offer high quality education for all learners, the teachers and the organization need to improve diversity management in order to build the human capital potential required to fulfill such tasks and meet educational challenges. Good organization/school management may minimize the negative effect of an additional burden on all the constituents of the mainstream educational environment [11].

The concept of diversity management is usually associated with the differences between employees in an organization arising from their respective personalities, cultures, religious affiliations or socio-economic status [12] and the possible benefits of this diversity for the effectiveness of the organization [13,14]. It seems a very good idea to use this concept in analyses of the specificity of inclusive educational institutions, where human capital comprises not only the diversity of personnel but also the diversity of pupils (abled/disabled) and of the significant others (parents of abled/disabled pupils). The results of existing analyses clearly suggest that such diversity may be beneficial for an organization/school [13] as well as for every individual that comprises its human capital [15,16,17,18].

The tradition of analyzing an educational institution as an organization is long-established [19,20,21]. There exist a number of studies that focus on analyzing the climate of an organization (school) [22,23,24,25]. In their historical review, Zullig et al. ([26], p. 140) noted that although school climate was for the first time mentioned in the early 20th century, scientific research started only 50 years later. Since then, numerous researchers have on the one hand highlighted the determinants of organization/school climate and on the other hand explained a number of phenomena in the contemporary school by positive/negative evaluation of that climate, especially phenomena involving the teachers [9,27,28,29,30,31], pupils [25,32,33,34,35,36,37,38,39] and parents [40,41]. The importance of organizational climate for the effectiveness of an inclusive educational institution is also noted increasingly often [42,43,44].

As already mentioned, the organizational/school climate is a theoretical construct used to analyze the quality of functioning of an educational institution as an organization. Based on the knowledge of the organizational climate [45,46], various researchers seek to translate the concept and measurement methodologies into descriptions of school or classroom climate [19,22,24,25,26]. In the literature on the subject, we can find a whole range of theoretical deliberations whose authors review the theoretical concepts of measuring organizational climate. The overall conclusion of those reviews is that the existing concepts may be grouped on the basis of the criterion of: objective evaluation of indicators/subjective evaluation of organizational/school climate by employees, pupils, and parents [46]. One of the most frequently used concepts of organizational climate as a subjective category is that developed by [45], according to which the organizational climate incorporates a set of measurable properties of a working environment as they are directly or indirectly perceived by the individuals who live and work in that environment, which affects their motivation and behavior. The multiplicity of concepts translates into a multiplicity of tools for studying the organizational climate. The research reported in this paper uses a tool that is consistent with Litwin and Stringer’s concept, incorporating the OCE methodology and adapted to the Polish context by Wudarzewski ([47], pp. 347–353). The tool describes six determinants of organizational climate: flexibility; accountability; work standards; rewards; clarity (comprehensibility) of the mission and procedures; and team engagement.

Even though, from the perspective of the organization’s inherent occupational factors affecting the level of teacher/educator’s stress, the most comprehensive approach is an analysis of subjective perception of the organizational climate, the most comprehensive approach from the perspective of personal determinants seems to be the measurement of job satisfaction and emotions accompanying work [48].

Generally speaking, job contentment is an attitude. It means either the inner state or the individual’s impression of how good or bad the work they do is for them. Currently, job satisfaction measurements and analyses focus on two corresponding components: its emotional and cognitive aspects. What we refer to as job satisfaction constitutes the cognitive aspects of being content with one’s work. Emotional aspects are the emotional evaluation of work, one’s mood or frame of mind at work [49]). This understanding of job satisfaction is consistent with the Transactional Model of Subjective Well-being proposed by Zalewska [50], which treats job satisfaction as a category of overall life satisfaction. This model emphasizes the distinctiveness of the respective emotional and cognitive evaluation and assumes that the qualities (resources) of a person modify the significance of inner and outer factors as well as the processing of emotional and cognitive information ([51], p. 2). 

Job satisfaction, which is believed to be a predictor of job performance [52], is increasingly used to assess job satisfaction among teachers/educators in mainstream schools [28,30,53,54] and special schools [55]. In some studies, the links between organizational climate and teacher job satisfaction [31,48,56] and pupil satisfaction are also examined [39].

Based on a literature review, Mielniczuk and Łaguna ([57] p. 2) have observed that “an opinion had long prevailed that emotions and rational thinking and acting are mutually exclusive. That is why researchers did not see emotions as an important factor potentially related to job performance. Nowadays, however, the interest in affect in the organizational context is increasing as evidence grows that emotional reactions are connected with rational decision-making, health, and different work outcomes”. Accordingly, new research reports are published that explain the share of emotions in job performance and describe the effects they bring about [58,59,60,61,62]. 

Of the many concepts and methodologies for measuring work affect, the most frequently used is the job-related affective well-being measure designed by Warr to assess four types of work affect: anxiety, comfort, depression, and enthusiasm [63]. The tool for describing emotions experienced in the workplace has been extensively modified by an international team led by Laguna [57,64,65,66]. The results of their work prove that the model used in the studies discussed herein, namely the model with four correlated factors, representing anxiety, comfort, depression, and enthusiasm, had a superior fit compared to alternative models and that mean scores on the scales of the instrument can be meaningfully compared across genders, but not across countries.

Summing up, it should be emphasized that every educational establishment providing high-quality education for all students should organize its activities around the current state of knowledge of diversity management. Diversity is found among the pupils (some have disabilities, others do not), the teachers (general education/special needs education teachers), and the parents (of typical pupils and those with disabilities), who altogether constitute the educational environment. From the organization theory perspective, a mainstream educational establishment is markedly different from a special school where such a wide diversity of stakeholders is not observed and which does not require the knowledge of diversity management [9,10]. Given that, this research adopted the type of an educational establishment (a mainstream school or special school) as the potential moderator of the correlation between an organizational climate and teachers’ job satisfaction and work affect

The following research question was asked: To what extend does the type of an educational institution (mainstream or special school) serve as a moderator for the correlation between subjective evaluation of organizational climate and teacher job satisfaction and emotions their work triggers in teachers? Based on the state of knowledge, the following hypotheses were also made:

**Hypothesis** **1** **(H1).** *The type of an educational institution statistically significantly differentiates the analyzed variables (organizational climate, job satisfaction, work affect).*

**Hypothesis** **2** **(H2).** *The organizational climate has a positive, statistically significant correlation with job satisfaction and positive work affect, and a negative correlation with negative work affect*.

**Hypothesis** **3** **(H3).** *Organizational climate is a strong predictor of job satisfaction and work affect*.

**Hypothesis** **4** **(H4).** *The type of an educational institution is a moderator of the correlation between organizational climate and life satisfaction and work affect*.

## 2. Method

### 2.1. Participants

The study involved 214 mostly female teachers, half of the sample came from mainstream schools and the other half from special schools at different levels (primary schools, middle schools, high schools). The respondents represented various age groups, from beginner teachers to teachers heading for retirement age, and the mean ages were, respectively: for mainstream schoolteachers—*M* = 45.40, for special schoolteachers—*M* = 46.30. The average length of employment was ca. 20 years. The two groups were homogeneous in terms of the demographic characteristics (Table 1 and Table 2). Because of the special organization of work in the mainstream schools, ca. 50% of the teachers from mainstream schools did not work with pupils with special educational needs during the study (Table 1).

### 2.2. Research Tools

The Organizational Climate Questionnaire—adapted by Wudarzewski [47]—evaluates the perception of an organization’s climate by its members in general terms and with respect to six specific dimensions consisting of 14 items. The dimensions are called: flexibility—openness to new ideas (e.g., “Employees in my team are encouraged to solve problems on their own and to be more independent at work”); accountability—autonomy, the degree of employee independence (e.g., “Taking risks is a good approach to being a successful member of my team”); standards—the manager’s insistence on doing a good job (e.g., “My team makes sure to maintain high performance standards”); rewards—the existence and scope of positive incentives (e.g., “My team recognizes and appreciates consistently good performance”); clarity—uniform understanding of the goals, tasks, principles and procedures (e.g., “Employees in my team know the vision, assumptions, goals, tasks, and the rules of functioning in our organization”); team engagement—a sense of cooperation and shared responsibility for the work done (e.g., “Employees in my team trust each other”). All 14 items are evaluated on a Likert-type scale, where one means that the respondent does not agree with the described situation and that the situation is not relevant to their organization (school), and 6 that the respondent agrees with the described situation and that the situation is relevant their organization (school). Only the overall results are converted to sten scores; the results for the particular dimensions are presented as arithmetic means, which can range from one to six. The internal reliability of the questionnaire is high—Cronbach’s alpha for a heterogeneous group and the entire OCE scale is 0.95; its values for individual dimensions are 0.82 for flexibility, 0.75 for accountability; 0.85 for standards, 0.92 for rewards, 0.73 for clarity; and 0.87 for team engagement.

As already mentioned, teacher job satisfaction was evaluated in terms of two aspects: cognitive and emotional. This approach necessitated using two tools: the Satisfaction With Job Scale, by M. Zalewska [49] and the Work Affect Scale. The Satisfaction With Job Scale [49] measures the cognitive aspect of overall job satisfaction. It consists of 5 statements (e.g., “I have so far been successful in achieving at work what I intended to achieve”) rated on a 7-level scale, where 1 means—I strongly disagree and 7 means—I strongly agree. The outcome is calculated by adding the scores for the 5 items and can range from 5 to 35; the higher the score, the higher the job satisfaction. The internal reliability of the scale is high—in the heterogeneous group Cronbach’s Alpha, it is 0.86 (in four groups: 0.81–0.88). The scale has a one-dimensional structure: Factor analysis (principal components) yields one component with an eigenvalue exceeding unity, which accounts for 65.5% of the total variance in the whole group (in four groups: 58.5–69.8%). The scale shows high convergent validity with other measures of the cognitive aspect of job satisfaction and discriminant validity in relation to the measures of the emotional aspects of job satisfaction and the cognitive aspect of overall life satisfaction [49].

The Work Affect Scale has been adapted to the Polish context by E. Mielniczuk and M. Łaguna [57]. The scale consists of twelve emotions that describe the respondent’s mood in their workplace. Six of them are positive emotions (quiet, pleased, relaxed, joyful, enthusiastic, optimistic), and the other six are negative emotions (tense, anxious, upset, downcast, somber, unhappy). The respondents are asked to rate the frequency with which they have experienced the emotions over the last few weeks on the Likert-type scale, where 1 means—never and 6—means always (the result for each emotion can range from 1 to 6). The ratings of the experienced emotions enable an overall evaluation of positive affect and negative work affect (determined based on the total score for 6 emotions, which can range from 6 to 36). At the next step, the results obtained for emotions are divided into four types of work affect; anxiety, comfort, depression, enthusiasm (the total score for each type of affect can range from 3 to 18).

### 2.3. The Statistics/Statistical Procedures Used

The SPSS program was used in the statistical analyses. The state of the analyzed variables in the respective groups was described using the mean, standard deviation and the distribution of frequency and percentages. The mean values in the respective responding groups were analyzed using the *t*-test test or the U Mann-Whitney test. To compare the percentage distributions between the groups, the chi-square test was applied. The Pearson’s r coefficient was used to analyze correlations. In order to verify the theoretical model of moderation, Andrew Hayes’ [67] macro PROCESS version 3.4.1 was applied.

## 3. Results

### 3.1. State of the Studied Variables and How They Differ Depending on the School Type

When analyzing the results obtained by the responding teachers for the organizational climate variable, we noticed that both the overall result and the results in the respective dimensions were high in both groups of teachers (Table 3). This meant that, according to the responding teachers, the climate of educational institutions is mainly characterized by:

- Openness to new ideas from the personnel and the implementation thereof;
- A high level of teacher independence and a sense of autonomy in making decisions and choices;
- High work standards associated with the fact that managers expect a high quality of work and the goals set for the teachers stimulate them to improve their work and achieve better results;
- Appreciable incentives at place, being noticed and rewarded at work, and a multitude of positive stimuli addressed to the personnel;
- Strong coherence of goals, principles, and procedures that are understandable to the teachers;
- A high sense of teamwork and work sharing, a climate of a community that eagerly assists every team member.

The type of an educational institution (mainstream or special school) does not differentiate the overall evaluation of school climate, but its differentiating effect on the accountability dimension is statistically significant (*p* = 0.03). Teachers in special schools tend to perceive their schools’ climate as giving them significantly more autonomy compared with teachers in mainstream schools (size effect of 0.16) (Table 4).

With regard to the job satisfaction variable, the job satisfaction of the responding teachers, whether from mainstream or special schools, was average. The mean values for the two groups are very similar: *M* = 25.69 and *M* = 25.41, respectively. The standard deviations suggest a slightly bigger scattering of the results for the mainstream schoolteachers, but the type of teachers’ school does not differentiate their job satisfaction (Table 3). In general, it seems that the responding teachers did not see their job as ‘near-perfect’, considered their working conditions to be average, and did not always manage to achieve their goals in the work they did. These observations somewhat distort the almost ideal picture of schools in terms of organizational climate evaluation.

An analysis of the respondents’ emotions about their work in mainstream or special schools showed that they were mostly positive (comfort, enthusiasm) rather than negative (anxiety, depression), and that the type of school did not have a significant differentiating effect (Table 3).

To sum up, it should be noted that the variables analyzed for the responding teacher groups clearly show that, according to the teachers, the organizational climate of schools that teach pupils with special educational needs is good for work, whereas their job satisfaction is average though filled with many positive emotions.

### 3.2. Correlations between Organizational Climate, Satisfaction and Emotions at Work

The matrix of correlations between the organizational climate, job satisfaction and work-related emotions shows that both the overall organizational climate score and the scores for its particular dimensions have moderate or high positive correlations with job satisfaction, as well as with the positive affect and its components: comfort and enthusiasm, while having low negative correlations with the negative affect and its components: anxiety and depression. All the correlation coefficients are statistically significant (45 at *p*= 0.01 and 4 at *p* = 0.05) (Table 5). 

At the same time, strong positive correlations have been found between the organizational climate, job satisfaction, and work-related emotions. The more positive opinion of the organizational climate teachers had, the higher their job satisfaction and the more positive emotions they experience at work.

To test the predictive power of job satisfaction, positive affect and negative affect based on the organizational climate, linear regression analysis was carried out. The linear model built to this end was found to be statistically significant *F*(1, 210) = 67.73; *p* = 0.001 and to explain 24% of the variance of the explained variable. Thus, the organizational climate is a statistically significant predictor of job satisfaction (*p* = 0.001), with the relationship between the two variables being positive and moderately strong (β = 0.49). The higher the organizational climate, the higher the job satisfaction (Table 6). 

The model also proved to be statistically significant *F*(1, 210) = 16.23; *p* = 0.001 and to account for 7% of the variance of the explained variable. Accordingly, the organizational climate is a statistically significant predictor of negative affect (*p* = 0.001); the relationship between these two variables is negative and weak (β = −0.27). The worse the organizational climate, the stronger the negative affect. It is also a statistically significant predictor of positive affect (*F*(1, 210) = 68.14; *p* < 0.001); in this case, the relationship is positive and moderately strong (β = 0.50). The higher the organizational climate, the higher the positive affect (Table 6).

The next step in the analysis was to verify whether the type of an educational institution moderates the relationship. The first moderation model proved to be statistically insignificant, *F*(1, 208) = 0.02; *p =* 0.90, coefficient B = −0.01 [CI: −0.11; 0.09]. This means that the type of school does not change either the strength or the direction of the relationship between organizational climate and job satisfaction (∆*R*^2^ = 0.001) (Table 7).

The second moderation model proved to be statistically insignificant, *F*(1, 208) = 2.98; *p* = 0.09, coefficient B = 0.09 [CI: −0.01; 0.20]. This means that the type of school does not change either the strength or the direction of the relationship between OCE and positive affect (∆*R*^2^ = 0.0103) (Table 7).

The third moderation model proved to be statistically insignificant, *F*(1, 208) = 0.002; *p* = 0.96, coefficient B = 0.003 [CI: −0.11; 0.11]. This means that the type of school does not change either the strength or the direction of the relationship between OCE and negative affect (∆*R*^2^ ≤ 0.0001) (Table 7).

To sum up, the results clearly suggest that teacher perception of organizational (school) climate is a strong predictor of their job satisfaction and the emotions triggered by their work.

## 4. Discussion

The goal of the reported studies was to empirically verify the hypotheses and to describe the predictive scope of subjective organizational climate evaluation on the level of job satisfaction and work affect among mainstream and special schoolteachers, and to determine the extent to which the type of an educational institution moderates the relationship between the studied variables. The results of the analyses confirmed the first hypothesis: The type of an educational institution statistically significantly differentiates the analyzed variables (organizational climate, job satisfaction, work affect) to a limited extent only. There were no statistically significant differences in the mean scores in the responding teacher groups for any of the analyzed variables. The only difference concerned the *Accountability dimension* of the organizational climate variable: special schoolteachers notice significantly more situations in their organizations/schools that encourage them to be accountable for their tasks and decisions. This only difference in the subjective perception of organizational climate between the studied groups of teachers may be due to a noticeably greater dynamics of the teaching process when all pupils in the classroom have special educational needs. Then, the organization of work and the need to carry out the teaching curriculum constantly necessitate making choices and decisions to ensure teaching effectiveness. 

The other two hypotheses (H2. The organizational climate has a positive, statistically significant correlation with job satisfaction and positive work affect, and a negative correlation with negative work affect, and H3. Organizational climate is a strong predictor of job satisfaction and work affect) were fully confirmed in the course of analysis. Its results clearly show that perceived organizational climate is a very strong predictor of job satisfaction and work affect for teachers in both mainstream schools and special schools.

The results are entirely in line with the existing state of knowledge, which suggests a strong predictive role of subjective evaluation of organizational climate on numerous individual and relational variables [24,30,31,48,68,69]. 

The consistency between the findings of this study on the mainstream and special schools’ teachers and the information collected so far should be a strong argument for people in charge of organization of work in mainstream schools and similar establishments to make a wider use of the knowledge of diversity management [11]. Because of the diversity of stakeholders in mainstream schools is especially wide, it seems necessary to urgently start research to find the most effective ways to manage diversity in these complex and unique institutions.

The last hypothesis: H4. The type of an educational institution is the moderator of the correlation between organizational climate and life satisfaction and work affect was not, unfortunately, confirmed by the analyses. The type of an educational institution (mainstream or special school) does not moderate the correlation between organizational climate and job satisfaction and work affect. The correlations are similar in both responding groups. 

Like all research projects, this one, too, has certain limitations. One of them is that it did not control for additional educational demands of pupils with special needs in mainstream schools. The spectrum of pupils with certified special educational needs is very wide, ranging from those with minor learning difficulties to those with severe functional disorders. This may generate additional challenges in the organization of the teaching process so that they benefitted pupils with and without disabilities.

## 5. Conclusions

Summing up, this study supports the assumption that a school’s organizational climate is a strong predictor of teachers’ job satisfaction and work-related emotions and rejects the assumption that the relationship is moderated by the type of the school. This is an important positive message for managers organizing high quality education for all and for stakeholders from communities surrounding educational institutions [70].

Proper diversity management in mainstream schools creates a good organizational climate (an organizational factor) that warrants high job satisfaction and the positiveness of teachers’ emotions about their job (an individual factor). The co-occurrence and interaction between these two factors are a prerequisite to a synergy effect translating into high quality education for all pupils.

## Figures and Tables

**Table 1:** Distribution of the frequency and percentages of selected characteristics of the responding teachers.

		Mainstream Schools		Special Schools	
		*f*	*p*	*f*	*p*
Gender	Female	93	86.90	93	86.90
	Male	14	13.10	14	13.10
Place of residence	Village	9	8.40	11	10.30
	Town	8	7.50	6	5.60
	City	90	84.10	90	84.10
Currently works with a pupil with special educational needs	Yes	54	50.50	107	100.00
	No	53	49.50	0	0.00

Frequency (*f*), percentages (*p*).

**Table 2:** Selected characteristics of the responding teachers.

	Mainstream School				Special Schools			
	*M*	*SD*	*Min*	*Max*	*M*	*SD*	*Min*	*Max*
Age	45.40	8.49	29	62	46.30	6.86	28	60
Seniority	19.83	8.79	4	40	21.38	7.00	4	38

Mean (*M*), standard deviation (*SD*), minimum (*Min*) and maximum (*Max*).

**Table 3:** Mean scores, standard deviation and differentiation of the analyzed variables between the respective groups.

	Mainstream Schools		Special Schools		Test of Significance	
	*M*	*SD*	*M*	*SD*	*t*	*p*
Organizational climate	64.99	12.07	66.40	11.74	−0.87	0.39
Job satisfaction	25.69	5.21	25.41	4.56	0.42	0.68
**Positive Affect**	24.32	4.94	23.09	5.57	1.71	0.09
Comfort	11.78	2.60	11.31	2.76	1.25	0.21
Enthusiasm	12.54	2.66	11.77	3.13	1.93	0.054
**Negative Affect**	12.64	4.12	13.59	5.08	−1.51	0.13
Anxiety	7.38	2.50	7.92	3.06	−1.41	0.16
Depression	5.25	2.01	5.67	2.41	−1.36	0.18

Mean *(M*); standard deviation (*SD*); *t*—dependent samples test statistics; *p*—statistical significance.

**Table 4:** Comparison of the dimensions of organizational climate between the respective groups.

Type of School					
	Mainstream Schools	Special Schools	Comparison Test		Size Effect
	Mean Rank	Mean Rank	*U* Mann-Whitney	*p*	*r*
Flexibility	108.81	104.15	5370.5	0.57	-
Accountability	97.89	115.27	4696.5	0.03	0.16
Standards	101.37	111.72	5069.0	0.21	-
Rewards	106.88	106.11	5576.0	0.93	-
Clarity	103.30	109.76	5275.5	0.44	-
Team engagement	99.55	113.58	4874.0	0.09	-

*p*—statistical significance; *r*—size effect.

**Table 5:** Correlation coefficients between organizational climate, job satisfaction and positive and negative affect.

	*JS*	*AfPos*	*AfNeg*	*Anx*	*Comf*	*Depr*	*Enth*
*OCE*	0.49 **	0.50 **	−0.27 **	−0.26 **	0.45 **	−0.23 **	0.48 **
*OCE/F*	0.50 **	0.49 **	−0.29 **	−0.25 **	0.44 **	−0.29 **	0.48 **
*OCE/A*	0.48 **	0.45 **	−0.19 *	−0.19 **	0.40 **	−0.15 *	0.45 **
*OCE/S*	0.44 **	0.42 **	−0.23 **	−0.20 **	0.37 **	−0.22 **	0.42 **
*OCE/R*	0.54 **	0.49 **	−0.29 **	−0.28 **	0.46 **	−0.26 **	0.47 **
*OCE/C*	0.44 **	0.44 **	−0.21 **	−0.21 **	0.38 **	−0.19 *	0.44 **
*OCE/TM*	0.33 **	0.37 **	−0.22 **	−0.23 **	0.36 **	−0.16 *	0.34 **

* *p* < 0.05; ** *p* < 0.01; OCE—organizational climate; OCE/F—Flexibility; OCE/A—Accountability; OCE/S—Standards; OCE/R—Rewards; OCE/C—Clarity; OCE/TM—Team engagement; JS—Job satisfaction; AfNeg—Negative affect; AfPos—Positive affect; Anx—Anxiety; Comf—Comfort; Depr—Depression; Enth—Enthusiasm.

**Table 6:** Explanatory variables for job satisfaction and positive and negative affect.

	**Job Satisfaction**		
	***Beta***	***t***	***p***
OCE	0.49	8.23	0.001
*R* = 0.49; *R*^2^ = 0.24; *Corrected R*^2^ = 0.24; *F*(1, 210) = 67.73; *p* = 0.001			
	**Negative Affect**		
	***Beta***	***t***	***p***
OCE	−0.27	−4.03	0.001
*R* = 0.27; *R*^2^ = 0.07; *Corrected R*^2^ = 0.07; *F*(1, 210) = 16.23; *p* = 0.001			
	**Positive Affect**		
	***Beta***	***t***	***p***
OCE	0.50	8.25	0.001
*R* = 0.50; *R*^2^ = 0.25; *Corrected R*^2^ = 0.24; *F*(1, 210) = 68.14; *p* = 0.001			

OCE—organizational climate.

**Table 7:** Moderation effect for the analyzed relationships.

	**Job Satisfaction**								
						**Interaction**			
	**B**	**SE**	***t***	***p***	**CI**	**b1**	**p1**	**b2**	**p2**
OCE	0.22	5.08	2.81	0.005	0.07; 0.38				
TofS	−0.26	3.43	−0.08	0.939	−7.02; 6.50				
OCExTofS	−0.07	0.05	−0.13	0.898	−0.11; 0.09	-	-	-	-
Constant	12.06	5.22	2.31	0.022	1.78; 22.34				
	*R*^2^ = 0.2490; *F*(3, 208) = 22.98; *p* < 0.005					∆*R*^2^ = 0.0001; *F*(1, 208) = 0.02; *p* = 0.898			
	**Positive Affect**								
						**Interaction**			
	**B**	**SE**	***t***	***p***	**CI**	**b1**	**p1**	**b2**	**p2**
OCE	0.10	0.08	1.16	0.246	−0.07; 0.26				
TofS	−7.85	3.63	−2.17	0.032	−15.00; −0.70				
OCExTofS	0.09	0.05	1.73	0.086	−0.01; 0.20	-	-	-	-
Constant	19.84	5.52	3.60	0.001	8.97; 30.71				
	*R*^2^ = 0.2807; *F*(3, 208) = 27.06; *p* < 0.001					∆*R*^2^ = 0.0103; *F*(1, 208) = 2.98; *p* = 0.086			
	**Negative Affect**								
						**Interaction**			
	**B**	**SE**	***t***	***p***	**CI**	**b1**	**p1**	**b2**	**p2**
OCE	−0.12	0.08	−1.41	0.161	−0.28; 0.05				
TofS	1.00	3.58	0.28	0.780	−6.06; 8.05				
OCExTofS	0.01	0.05	0.05	0.961	−0.11; 0.11	-	-	-	-
Constant	18.95	5.44	3.48	0.001	8.21; 29.68				
	*R*^2^ = 0.0878; *F*(3, 208) = 6.67; *p* < 0.001					∆*R*^2^ = 0.0001; *F*(1, 208) = 0.01; *p* = 0.961			

## Data Availability

The data presented in this study are available on request from the corresponding author.
